# Natural Killer Cells as Helper Cells in Dendritic Cell Cancer Vaccines

**DOI:** 10.3389/fimmu.2015.00013

**Published:** 2015-01-28

**Authors:** María Betina Pampena, Estrella Mariel Levy

**Affiliations:** ^1^Centro de Investigaciones Oncológicas CIO-FUCA, Buenos Aires, Argentina

**Keywords:** cancer vaccines, natural killer cells, immunomonitoring, dendritic cells vaccines, NK cells–dendritic cells crosstalk

## Abstract

Vaccine-based cancer immunotherapy has generated highly variable clinical results due to differing methods of vaccine preparation and variation in patient populations among other lesser factors. Moreover, these clinical responses do not necessarily correspond with the induction of tumor-specific cytotoxic lymphocytes. Here, we review the participation of natural killer (NK) cells as alternative immune components that could cooperate in successful vaccination treatment. NK cells have been described as helper cells in dendritic cell-based cancer vaccines, but the role in other kinds of vaccination strategies (whole cells, peptide, or DNA-based vaccines) is poorly understood. In this article, we address the following issues regarding the role of NK cells in cancer vaccines: NK cell anti-tumor action sites, and the loci of NK cell interaction with other immune cells; descriptions of new data on the memory characteristics of NK cells described in infectious diseases; and finally phenotypical and functional changes after vaccination measured by immunomonitoring in preclinical and clinical settings.

## Introduction

Within the lymphocyte gate in the flow cytometric analyzer, natural killer (NK) cells are usually defined as CD3^−^ (thereby excluding T cells) and CD56^+^, an isoform of the neural cell adhesion molecule (NCAM) (1). NK cells constitute about 5–20% of peripheral blood (PB) mononuclear cells and are also found in many tissues such as the liver, peritoneal cavity, placenta, uterine mucosa, and lungs (2–6). Approximately, 90% of PB and spleen NK cells are CD56^dim^CD16^+^ and are characterized by a potent cytotoxic activity after interaction with target cells. On the other hand, NK cells in lymph nodes and tonsils are mostly CD56^bright^CD16^dim/−^ and have a helper role in the production of IFN-γ in response to IL-12, IL-15, IL-18, and type I IFN stimulation (1, 6, 7). Unlike T and B cells, NK cells have the unique ability to kill transformed or virally infected cells without prior sensitization. Furthermore, NK cells are rapidly recruited to the sites of virus entry and are critical for the control of acute viral infections (8, 9). In fact, individuals with NK cell deficiency suffer recurrent viral infection as a result of their impaired ability to develop lasting and effective antigen (Ag)-specific recall responses (8, 9). Moreover, NK cells can eliminate tumor cells, as has been shown both *in vivo* and *in vitro* (10, 11). NK cells spare healthy cells that express MHC class I molecules and low levels of stress-induced self-molecules, but are capable of recognizing and directly killing a wide variety of tumor or virally infected cells with reduced levels of MHC class I molecules or that overexpress stress-induced activating cell surface molecules (e.g., MICA/B recognition via NKG2D) that may otherwise escape immune detection. These are known as the “missing-self” and “non-self” phenomenon, respectively (12). Additionally, NK cells are involved in the immune response against tumor metastasis (13). For instance, in a mouse model of metastatic lung cancer, authors found that NK cells prevented pulmonary metastasis and peritoneal dissemination following treatment with cationic liposomes complexes formed by CpG DNA (14). Another mouse model of lung metastases showed that NK cell depletion abolished the protective effect of IFN-γ treatment on metastases. In fact, there was crosstalk between NK cells and tumor cells through the IFN-γ-induced transcription factor IRF-1, which is expressed on tumor cells, supporting the pulmonary attraction and activation of NK cells (15). Direct tumor cell lysis by NK cells is thought to be mediated principally by perforins, as shown *in vivo* using experimental models of metastases in mice (16, 17). However, NK subset depletion resulted in more instances of metastases than observed in perforin-deficient mice, suggesting that the perforin-independent effector functions of NK cells may also contribute to protection from tumor metastasis. Moreover, NK cells can also induce tumor cell elimination through death receptor-mediated pathways such as TRAIL and FasL (18–20). On the other hand, activated NK cells are also potent producers of numerous immunomodulatory cytokines, including IFN-γ, TNF-α, growth factors such as G-CSF and GM-CSF, and numerous chemokines (21).

In humans, NK cells play an important role in tumor immunosurveillance alongside specific T lymphocytes. In an 11-year follow-up survey of a Japanese cohort study, it has been shown that low peripheral NK cell activity is associated with increased cancer risk (22). Other clinical studies have provided evidence that in several different solid tumors, such as lung, gastric, colorectal, and head and neck cancers, the presence of high numbers of tumor-infiltrating NK cells correlates with improved prognosis of cancer patients (pts) (23, 24). Moreover, decreased NK cell activity was observed in pts with hereditary colorectal adenocarcinoma (25, 26); and melanoma pts with metastatic disease have an impaired perforin-dependent NK cell cytotoxic mechanism (27). Menard et al. demonstrated the relevance of NK cells in gastrointestinal stromal tumor-bearing pts treated with imatinib mesylate (a tyrosine-kinase inhibitor). Apparently, those patients whose NK cell IFN-γ values were higher than or equal to their trial-entry baseline value after 2 months of therapy had prolonged disease-free survival compared to the others pts (28).

Considering the important role that NK cells have an immunosurveillance, it is desirable to focus the development of cancer therapies to augment NK cell killing and helping efficacy because it could aid in the induction of an optimal adaptive immune response against cancer.

## NK Cell Localization, Trafficking, and the NK Cell Detection Issue

Even though NK cells seem to be critical immune effectors in tumor cell elimination in *in vitro* experiments and animal models, they have a limited capacity to traffic to tumor sites. Of note, in humans, factors regulating NK cell recruitment into neoplastic tissues are highly influenced by the tumor type and by the chemokine profile of the tumor microenvironment. A recent study suggested that CD56^+^ NK cells could scarcely infiltrate melanomas, hepatocellular carcinomas, breast cancers, and renal cell carcinomas (29). Other studies reported that in solid tumors, NK cells are often located within the stroma area, not in direct contact with tumor cells, and are usually functionally anergic (30, 31). However, in this setting, it is difficult to establish whether these NK cells are activated (high CD56) NK cells that lost perforin expression through degranulation, or if they constitute an altered NK cell phenotype induced by the tumor cells.

A more recent study found that NK cells were widely distributed in most solid normal and neoplastic tissues and that the relative proportion of NK subsets infiltrating was different upon malignant transformation, with a trend toward a tumor-infiltrating NK population enriched in non-cytotoxic cells (6). Moreover, NK cells from melanoma metastatic lymph nodes were found surrounding tumor cell clusters and although they were mostly CD56^bright^ and inactive, they could be activated *ex vivo* by IL-2 or IL-15 and could lyse metastatic melanoma cells more efficiently than blood-derived NK cells (32). The appropriate activation of this NK cell subset could unfold their helper function, thereby turning T cell activation toward a TH_1_ response.

However, the apparent limited capacity of NK cell trafficking to tumor sites could be an artifact of the NK cell detection techniques used. NK cell detection methods are still a source of discussion because of doubts about the accuracy of the antibodies and molecular targets used in NK cell tissue-associated detection. Of note are the substantial differences when compared with tissue detection of T CD4 and T CD8 cells, which have good and reliable antibodies for immunohistochemistry.

As illustrated in Table 1, NK cell detection depends on the technique and molecular target used. There are several papers showing that NKp46 presents important advantages over other NK cell markers, such as CD56 or CD57, in the identification of human NK cells by immunohistochemistry. Moreover, NKp46 is commonly used for NK cell detection by flow cytometry and RT-PCR.

**Table 1:** **Detection of NK cells in different normal or neoplastic tissues by technique and molecular target**.

Method/technique	Target molecule	NK cell presence	Human normal or neoplastic tissue	Reference
Immunohistochemistry	CD57 (c NK-1)	Yes	Squamous cell lung cancer	(24)
	CD56 (c 123C3)	No or almost undetectable	Melanoma Hepatocellular carcinoma Breast cancer	(29, 33)
			Renal cell carcinoma	(34)
	CD56 (c 123C3)	Yes	Breast cancer	(35)
	CD56 (ns)	Yes	Melanoma	(36)
	CD57 (c NC1)	Yes	Gastric cancer	(37)
	NKp46 (c 195314)	Scarce	Colorectal cancer	(30)
		Yes	Lung, breast and colon (normal and tumor tissue)	(6)
			Metastatic melanoma lymph nodes	(32)
			Non-small cell lung cancer	(38)
Immunofluorescence	NKp46 (polyclonal), CD56 (c 123C3)	Yes	Spleen, gut and colon	(39)
RT-PCR	Specific differentially methylated regions near NKp46 gene	Yes	Leukocytes from peripheral blood from head and neck cancer pts	(40)
Flow cytometry (from disaggregated tissue)	CD56 (c AF12-7H3), NKp46 (c 9E2)	Yes	Lung, breast and colon (normal and tumor tissue) Gut and colon tissue Colorectal cancer Breast cancer	(31) (39) (41) (42)

*c, clone; ns, not specified*.

## NK–Dendritic Cell Crosstalk

Altogether, it is well accepted that NK cells possess potent anti-tumor functions that could be targeted for immune-based therapies (43–46). NK cell direct killing of target cells also impacts T cell responses, possibly by decreasing the antigenic load (47) and because target cell debris may promote Ag cross-presentation to CD8^+^ cytotoxic T cells (48). However, these direct anti-tumor effects can be attributed not only to cytotoxicity but also to their cytokine-producing capacities (IFN-γ, TNF-α, IL-10). Because NK cells can also indirectly contribute to tumor control by communicating with other immune cells (e.g., dendritic cells – DCs, NKT cells, macrophages, and T cells), there is an efficient adaptive anti-tumor response (12, 21, 49). Furthermore, it is well known from *in vitro* studies and colocalization experiments that NK cells can interact bidirectionally with DCs in areas of inflammation causing DC maturation, a consequent enhancement of NK cell function through positive feedback and exerting an influence on the polarization of primary T cell-responses toward a TH_1_ response (50–55). In fact, mature DCs can activate NK cell cytotoxicity and IFN-γ production, whereas activated NK cells are capable of enhancing DC maturation and IL-12 production. The previously described interactions are cell contact and TNF-α-dependent (50, 56–58). Furthermore, mature DCs recruit NK cells to the lymph nodes, where NK cells serve as an early source of the IFN-γ necessary for TH_1_ polarization, possibly by direct interaction with naïve T cells (54).

On the other hand, it has been shown that activated NK cells can kill autologous immature DCs, while they spare fully activated DCs. This work lead to the proposal that activated NK cells might select a more immunogenic subset of DCs during a protective immune response (58, 59). Interestingly, in a recent mouse model of vaccination against breast cancer, authors showed that the addition of YAC-1 (a NK target cell devoid of MHC) in a vaccine composed of irradiated tumor cells boosted the expansion of tumor-specific cytotoxic T lymphocytes, eventually resulting in enhanced survival of mice upon challenge with a lethal dose of tumor cells. NK cells removed immature tolerogenic DCs and the residual DCs were highly immunogenic. These DCs could induce proper T cell clonal expansion, which gave anti-tumor protection. The depletion of NK cells impaired the tumor-specific T cell response and, consequently, their protective roles upon tumor challenge (60). In a more recent tumor model, Bouwer et al. found that *in vivo* depletion of NK cells at the time of tumor challenge completely abolished the benefit of bacteria-stimulated DC immunotherapy. Although CD4^+^ or CD8^+^ T cells may be required for an optimal anti-tumor response, the loss of NK cells resulted in a more profound defect in tumor immunity. They also found that NK cells exert a helper role in priming and reactivating tumor-specific T cells because the contribution of NK cells was dependent on tumor-Ag presentation by DCs. However, unlike the previous work, the contribution of NK cells in the context of this vaccination did not rely on the perforin-dependent lysis of tolerogenic DCs in draining lymph nodes because IFN-γ, not perforin, was essential for the success of DC immunotherapy (61).

Since NK cells cooperate with DCs and T cells to enhance anti-tumor responses, cancer vaccines could be improved by strategies aimed at activating NK cells. There is a rationale for NK cell immunomonitoring in cancer immunotherapeutic approaches. However, although it is clear that effector cells are the main targets of immunotherapy, treatments should also focus on immune cell trafficking to tumor sites. The knowledge that tumors disrupt T and NK cell homing through different mechanisms is useful for the implementation of combination immunotherapies to overcome these immunosuppressive mechanisms (62).

## NK Cell Memory

In recent years, a new role for NK cells has been described. Under certain experimental conditions, NK cells share some features with adaptive immune cells, such as the Ag-dependent expansion observed in mice infected with murine cytomegalovirus. This NK cell subset expansion is associated with long-lasting functional changes with features similar to memory T cell populations (63). There is another mouse model in which the challenge is made by hapten-induced contact hypersensitivity. Work in this model demonstrated that these memory–NK cells are confined to the liver, since hapten-specific memory is conferred to naïve mice by adoptive transfer only of liver NK cells from sensitized donors (64, 65). In another work, the authors show that *in vitro* cytokine-activated NK cells transferred into naïve recipients can persist for at least a month. Although they are phenotypically similar to naïve cells and do not constitutively produce IFN-γ, these memory-like NK cells produce significantly more IFN-γ when restimulated, displaying an intrinsic capacity to respond more robustly after reactivation with cytokines or via engagement of activating NK receptors (66). However, Horowitz et al. demonstrated in an experimental model of a rabies virus vaccine, that there are no intrinsic differences between prevaccination and post-vaccination NK cells, although the last cells to respond have a more robust response. In fact, post-vaccination NK cells are simply responding to IL-2 produced by memory CD4^+^ T cells and IL-12 and IL-18 produced by accessory cells after virus rechallenge (67).

Nevertheless, there is a lack of definitive evidence for NK cell memory in humans. Study suggests that infection with human cytomegalovirus skews the NK cell receptor repertoire toward the activating CD94/NKG2C receptor that is usually expressed on <10% of total NK cells in PB (68). After expansion, the NKG2C^+^ NK cells were more potent producers of IFN-γ than their NKG2C counterparts and expressed CD57, a marker of terminal differentiation (69). These findings suggest that once NK cells are activated, they acquire certain characteristics that influence their behavior during subsequent encounters with Ags.

## Immunomonitoring

The primary objective of immune monitoring in cancer vaccine clinical trials is to find a correlation between an efficient induction of tumor-specific T cell responses and clinical efficacy that reflects the importance of the host immune system in controlling tumor progression. However, although there is evidence of increased frequency of tumor-specific T cells in several cancer vaccine trials, no validated biomarkers exist yet for cancer immunotherapy. As mentioned above, NK cells cooperate with T cells to enhance anti-tumor response and this emphasizes the importance of optimizing NK cell activation for tumor immunotherapeutic protocols. NK cell monitoring was studied more frequently in tumor-Ag-loaded DC immunization trials (Table 2). The range of vaccines developed has included peptide/protein-based vaccines, cell lysates, and whole-tumor cell vaccines with different delivery systems and adjuvants (70). In practice, though, only a few groups have implemented the evaluation of NK cells in clinical trials. Table 2 provides an overview of DC-based tumor vaccination trials implementing NK cell monitoring. The main NK cell activities include IFN-γ production and lytic activity against the K562 cell line. High IFN-γ production is an indicator of resident immunostimulatory NK cells, which is especially interesting in light of DC-based approaches. Other cytokines produced by NK cells, e.g., TNF-α, GM-CSF, IL-10, and IL-13, may be considered for the evaluation of tumor cell sensitivity to NK cell-mediated killing and could be significant predictive markers for therapy effectiveness (71). Moreover, NK cells are defined by their intrinsic capacity to kill transformed cells and in this sense, their activation could be evaluated as the capacity to degranulate, produce granzymes or perforins, and lyse tumor target cells or their canonical target, the K562 cell line. However, it is important to note that cytotoxicity against the universal target K562 may not correlate to cytotoxicity against patient tumor cells.

**Table 2:** **NK cell immunomonitoring from different clinical trials using dendritic cell cancer vaccines**.

Protocol	Vaccine type	Tumor type	NK n° and phenotypic changes	NK cells lysis	NK cells cytokines production	Association with pts outcome	Reference
Dose-escalation phase I trial	Autologous DCs transfected with an adenovirus encoding IL-12 gene	Metastatic gastrointestinal cancer	Not evaluated	↑Cytotoxic activity vs. K562 cells in 5/15 pts after treatment	↑IFN-γ and ↑ cytotoxic in 4/15 pts after treatment.	From pts with ↑cytotoxic NK cell activity: 1/5 patients achieved a PR and 1/5 experienced a clear SD. 3/4 pts with PD had a transient ↑cytotoxic activity	(72)
Phase I trial	Autologous DCs loaded with a fowlpox vector encoding CEA	CRC, lung cancer and urachal adenocarcinoma	5/9 pts ↑NK cell n° during vaccination, 2 did not change and 2 ↓NK cell n°. ↓NKG2A in 2/5 pts; ↑NKG2D in 3/5 pts. NKp46 and NKG2D expression correlated with activity	4/9 pts ↑NK cell activity (3 of them had also ↑ NK cell numbers). 2/9 stable NK cell activity, and 3/9 ↓NK cell activity	Not evaluated	4 of 5 SD/NED pts had ↑ NK cell activity	(73)
Pilot trial	DCs loaded with autologous HS- and UV-C−treated tumor cells	FL B cell NHL and lymphoplasmocytoid lymphoma	↑CD3^−^CD56^dim^CD16^+^ and ↑CD16 MFI ratios and ↑NKp46 after treatment	Not evaluated	Not evaluated	↑CD3^−^CD56^dim^CD16^+^ in R compared with NR pts. ↑ NKp46 4/6 R pts in comparison with 1/4 NR pts with a similar change	(74)
Phase I/II trial	Monocyte-derived WT1 mRNA-loaded DC	AML	↑HLADR^+^ NK cells in pts after treatment	Not evaluated	Not evaluated	Correlation between CR and ↑of activated NK cells post-vaccination (i.e., more than 40% HLA-DR + cells within the total NK cell population in 4/5 CR and 0/5 NR)	(75)
Pilot trial	Autologous DC loaded with autologous tumor lysates preheated + pre-treatment with CTX + PegIFN	HCC, ChC, CRC, Carc, M	6/17 pts ↑% of NK cells modestly	↑Cytotoxic activity against K562 cells after first cycle (11/17 pts) and after second cycle (8/17 pts)	Not evaluated	No clinical correlates with immune and biological parameters observed	(76)
Phase I/II trial	Autologous DCs + IL-2	Renal cell carcinoma and BC	2/6 pts ↑CD16^+^CD56^dim^	6/10 pts ↑cytotoxic activity vs. K562 cells	Not evaluated	Only one patient with objective CR, associated with CD8^+^ IFNγ production	(77)
Phase I	DCs loaded with autologous tumor lysates	ER^−^/PR^−^stage II/IIIA BC	↑n° of NK cells	Not evaluated	Not evaluated	Not analyzed	(78)
Phase I	DC pulsing with autologous tumor cell lysates	Recurrent Glioblastoma	6/15 pts ↑% of NK cells, which further augmented after the second vaccination	Not evaluated	↑IFNγ associated with a ↑% of NK cells after the first vaccination	Pts with ↑NK V/B ratio had longer PFS and OS. And pts with ↑TGFβ2 and VEGF V/B ratios had a shorter PFS and OS	(79)

*n°, numbers; ↑, increase; ↓, decrease; CR, clinical responders; FL, follicular; NHL, non-Hodgkin lymphomas; lymphoplasmocytoid lymphoma; R, responder; NR, non-responder; IL, interleukin; ITI, intratumoral injection; PR, partial response; SD, stable disease; NED, no evident disease; PFS, progression free survival; OS, overall survival; V/B, vaccination/baseline ratio; CTX, cyclophosphamide; PegIFN, PegIFN alpha-2a; HCC, hepatocellular carcinoma; BC, breast cancer; ChC, cholangiocarcinoma; CRC, colorectal carcinoma; Carc, carcinoid tumor; M, melanoma; HS, heat-shocked*.

Natural killer cell immunomonitoring in DC vaccine clinical trials showed a correlation between the clinical responses of treated pts and NK cell status. NK cell responses were analyzed in a phase I trial of a vaccine consisting of autologous DCs loaded with a fowlpox vector encoding CEA and the data were compared with pt clinical outcome. DCs enhanced NK activity *in vitro*, by both sustaining NK cell survival and by enhancing the expression of NK-activating receptors, including NKp46 and NKG2D. Of the nine pts, four had increased cytolytic NK activity, including three pts with increased NK cell frequency; this remained stable in two pts and decreased in three as compared to pre-treatment values. NKp46 and NKG2D expression were correlated with the pts’ NK cell activity. When pts were grouped by clinical response, the majority in the stable/NED (no evident disease) group had increased NK activity. Anti-CEA T cell response was enhanced in all of the nine pts analyzed, but it was not significantly different between groups. Thus, NK responses following DC vaccination may correlate more closely with clinical outcome than do T cell responses (73). In a study of ER/PR double-negative stage II/IIIA breast cancer pts vaccinated with autologous DCs pulsed with autologous tumor lysates, DC vaccines elicited TH_1_ cytokine secretion and increased the number of NK cells (78). In another immunization treatment with monocyte-derived DC incubated with preheated autologous tumor lysate and subsequently with IFN-γ, TNF-α, and polyinosinic:polycytidylic acid to attain type 1 maturation, treatment induced sustained, elevated IL-12 serum levels that correlated with the IL-12p70 output of cultured DC from each individual. NK activity in PB increased and was also correlated with the IL-12p70 serum concentration in each pt (76).

As mentioned above, NK cells were mostly studied in the context of antigen-loaded DC immunotherapy, and so far, we were not able to find another tumor vaccine study highlighting the importance of this lymphocyte subset. In our laboratory, we are testing an anti-melanoma vaccine composed by four irradiated melanoma cell lines plus BCG and GM-CSF as adjuvants against conventional IFN-α therapy. We have not seen changes in T CD4^+^ or T CD8^+^ cell frequencies post-treatment, although we did find a significant increase in NK cell number and frequency in vaccinated pts when comparing pre- and post-treatment samples. Studies are needed to find the cause of this NK cell number increase, and to see if this effect is correlated with pt clinical outcome (80).

Antibody-dependent cellular cytotoxicity (ADCC) is probably the most thoroughly evaluated activity performed by NK cells during treatment with monoclonal antibodies. NK cells can trigger ADCC to lyse IgG opsonized target cells. Several authors have reported a correlation between NK function and response to treatment with Cetuximab, Rituximab, and Trastuzumab in animal models and cancer pts (81, 82). Not only ADCC but also NK–CD interaction was observed in antibody treatments. For example, the interactions between Cetuximab with cancer cell EGFR and NK cell FcδR IIIa enhances cross-presentation of tumor Ags, such as EGFR by DC to cytotoxic T lymphocytes. In fact, there are more circulating EGFR-specific T cells in Cetuximab-treated head and neck cancer pts than in treatment naïve pts (83). This suggests that antibody administration could trigger a tumor-Ag-specific cellular immune response and could be combined with cancer vaccines to improve cancer immunotherapy.

In summary, although immunomonitoring demonstrated NK cell relevance in DC immunotherapeutic approaches and a correlation with pt response in several clinical trials, our understanding of their role remains incomplete. In fact, doubts remain about whether NK cells function primarily through tumor cytotoxicity or if NK cells also exert a relevant helper function through their interaction with DCs, T cells, and other immune cells. This second type of interaction remains the source of much discussion. Consequently, it is important to monitor NK cell numbers as well as phenotypic and functional changes, such as the ability to exert tumor cell lysis and the production of immunomodulatory cytokines during the course of immunotherapy. Furthermore, addressing the capacity of treatment to generate the kind of NK memory-cell described in the prior section (for example, measuring changes on NKG2C expression after treatment) could contribute to the long-term protection expected from cancer vaccines.

## Concluding Remarks

This review ventured beyond a description of the plurality of NK cell activities into the changes that therapeutic cancer vaccines can affect in NK cells, and how these lymphocytes can potentiate the immune system through DC vaccination. In this bidirectional crosstalk, NK cells hold the capacity to control and enhance DC-mediated anti-tumor immune responses by inducing the maturation of TH_1_-polarizing DCs, providing DCs with antigenic material for presentation and by killing inappropriately matured DCs. On the other hand, DCs stimulate NK cells by both soluble and contact-dependent activators, thereby enhancing their cytokine production, proliferation, survival, and cytotoxicity. Although this DC–NK cell interaction has been demonstrated *in vitro* and in animal models, a review of the literature found very few clinical trials that performed NK cells immunomonitoring. The majority of these DC cancer vaccine clinical trials showed a correlation between NK cell number/percentage and/or activity augmentation and pt outcome. Surprisingly, to the best of our knowledge, NK cell analysis has not been carried out in trials for other kinds of tumor vaccines. To gain more knowledge about the role of NK cells in immunotherapeutic cancer vaccines, NK cell monitoring must be systematically incorporated into clinical vaccination trials. This could lead to a better understanding of the real impact of NK cells in the vaccine field.

## Conflict of Interest Statement

The authors declare that the research was conducted in the absence of any commercial or financial relationships that could be construed as a potential conflict of interest.
